# A retrospective comparison of false negative skin test rates in penicillin allergy, using pencilloyl-poly-lysine and minor determinants or Penicillin G, followed by open challenge

**DOI:** 10.1186/s13223-015-0098-5

**Published:** 2015-11-20

**Authors:** Lana Rosenfield, Chrystyna Kalicinsky, Richard Warrington

**Affiliations:** Section of Clinical Immunology and Allergy, Department of Internal Medicine, Health Sciences Centre and University of Manitoba, Winnipeg, MB Canada

## Abstract

**Background:**

A history of penicillin allergy in patients is common, but only 10–15 % are truly allergic. While the gold standard for diagnosing penicillin allergy is challenge, it is not recommended that this be done without first carrying out diagnostic skin testing. This is carried out with the major determinant benzylpenicilloyl (PPL) and the minor determinant mixture (MDM), consisting of penilloate, penicilloate and Penicillin G. However, since availability of the MDM is limited, Penicillin G alone has been used.

**Methods:**

A retrospective chart review was carried out on patients tested for penicillin allergy in the Clinical Immunology and Allergy Clinic at the Health Sciences Centre, Winnipeg, Canada between 2005 and 2013. A total of 521 patients charts were reviewed, of whom 240 had skin testing, ImmunoCap^®^ for IgE to Penicillin G and V and had oral challenges with penicillin, amoxicillin or cloxacillin.

**Results:**

17/240 (7.5 %) were skin test positive, 8 to PPL, 4 to MDM and 5 to Penicillin G. One was also positive on ImmunoCap^®^ testing. Three patients had negative skin tests but weakly positive ImmunoCap^®^. 222 patients with negative skin tests and serological tests were challenged. Of these, 12 patients reacted to challenge. Three of the challenges were equivocal. Of the nine patients with definite positive challenges, three were tested with Penicillin G and six with MDM. Therefore the false negative rates for testing were 2.3 % with PPL and Penicillin G and 6.97 % for PPL and MDM. The difference was not significant (p = 0.0856).

**Conclusions:**

In this group of patients with a history of penicillin allergy tested with the major determinant of benzyl penicillin and either MDM or Penicillin G, there was no difference in the rate of false negative testing, based on oral penicillin challenges. Therefore, Penicillin G can be safely used as an alternative to MDM in diagnosing penicillin allergy.

## Background

Penicillin allergy is a common complaint of patients presenting to Allergy Clinics. Patients describe a variety of symptoms from immediate urticarial rash and respiratory symptoms including anaphylaxis, to more delayed reactions a few days after initiating the antibiotic or even after course completion [ [1, 2]. Previous studies have shown that patient histories of their reaction are not reliable to determine immediate or non-immediate reactions to penicillin [3, 4]. When diagnosing beta-lactam allergy it is possible to assess both immediate and delayed reactions [5–8]. Diagnosis of immediate drug allergy to beta lactam antibiotics is made by assessing history, skin testing, measurement of specific IgE levels and drug challenge [5, 9]. The gold standard for confirming IgE mediated allergy is reaction within 1 h after a challenge dose is given [8, 10, 11].

Skin testing utilizes both skin prick testing and intradermal tests [5, 9]. The major determinant Benzylpenicilloyl is the metabolized form of penicillin used in the form of benzyl penicilloyl-polylysine (PPL) for skin testing [12, 13]. The minor determinant mixture (MDM) consists of penicillin and products of penicillin metabolism, specifically penilloate and penicilloate. Availability of the MDM as well as PPL has been variable, depending particularly upon location. North America particularly has had limited access to both PPL and MDM. Using both major and minor determinants is considered the first line method for diagnostic skin testing for Beta-lactam immediate hypersensitivity [12, 14]. However, falling rates of reactivity to penicillin reagents have been reported [12, 15, 16]. Ampicillin, amoxicillin and cephalosporins can also be used for skin testing when reaction to a specific drug is in question and skin test reactivity to semi-synthetic penicillin may be now be more frequent [16, 17]. The usefulness of testing with the complete battery of minor determinants has been questioned [18, 19].

We undertook a retrospective chart review of patients who presented to the Allergy/Clinical Immunology clinic with suspected penicillin allergy and compared the efficacy of skin testing with PPL and MDM vs. PPL and penicillin alone, using open challenge as the gold standard for determining the presence or absence of penicillin allergy in those who were skin test negative.

## Methods

### Ethics

Ethics approval was obtained through the University of Manitoba Research Ethics Board and access to charts was obtained through Health Sciences Centre Department of Research.

### Patient selection

Charts of patients who had penicillin skin testing in the Allergy/Immunology Clinic at Health Sciences Centre in Winnipeg, Manitoba were reviewed. Patients who were skin tested between December 2005 and 2013 were included. Patient selection was done by searching electronic dictation letters and billing codes for penicillin and penicillin challenge, and charts were reviewed.

### Skin testing

All patients evaluated for penicillin allergy underwent skin prick testing, intradermal testing or both. Skin prick testing was done using a 27-gauge needle to prick the skin. For intradermal testing, 0.02 mL was injected. Reagent for the penicillin major determinant used was benzylpenicilloyl polylysine, in the form of Prepen (ALK-Abello, Canada), DAP (Diater, Spain), or laboratory made reagent, standardized by mass spectrometry). Penicillin minor antigenic determinants were a minor determinant mixture made in-house that included Penicillin G, penilloate and penicilloate at 2 × 10^2^ molar (characterized by mass spectrometry) [20], the DAP MDM mixture (concentration used was identical to the laboratory-made reagent) or Penicillin G alone, at a concentration of 6.2 mg/mL. The MDM used was determined by availability. Positive control for both intradermal and skin prick testing was a concentration of 1 mg/ml Histratrol (ALK-Abello). The negative control for skin prick test was glycerinated phenol saline (NaCl 0.9 %, glycerine 50 %, phenol 0.4 %) and sterile normal saline for intradermal testing. Ampicillin trihydrate at a concentration of 5 mg/mL was used if ampicillin or amoxicillin skin testing was required. Cloxacillin, and cephalosporins were used at the same concentration. All patients were tested with the major determinant and MDM or Pen G, depending on availability. Other testing was determined by patient history.

Skin testing was considered positive if there was a wheal 3 mm greater than negative control with flare at 15 min, for either major and minor determinants.

### ImmunoCap^®^

Blood samples from all patients were collected for ImmunoCap^®^ (Phadia, Sweden) testing to Penicillin G and V was done by the Clinical Chemistry laboratory at Health Sciences Centre in Winnipeg, Manitoba, Canada. A positive result is indicated by a value greater than 0.35 kU/L.

### Challenge

Patients with negative skin testing and negative penicillin V and G ImmunoCap^®^ were contacted to undergo challenge, which was done in the Allergy/Immunology Clinic at Health Sciences Centre using oral penicillin V 300 mg, amoxicillin 250 mg, Cloxacillin 250 mg, or Clavulin 250/125, depending on the original clinical precipitant. If not known, penicillin or amoxicillin was used. Patients were observed for 1 h, and were asked to contact the clinic if there was a delayed reaction, occurring later than 1 h. All patients met with their physician after the challenge. Patients with adverse events were assessed by the clinic physicians and outcomes, both positive and negative, were recorded.

## Results

521 patient charts were reviewed. Seventy-six patients did not have complete skin tests and serology. 205 patients did not return for challenge after negative skin tests and serology. Two hundred and forty patients had all investigations, including skin testing, ImmunoCap^®^ and challenge, or were diagnosed with penicillin allergy after one or more tests (skin test and ImmunoCap^®^). These 240 patients were used for the analysis. Of these 240 patients, there were 65 male and 175 female with a mean age of 46.7 years. Symptoms that had occurred with penicillin or other beta-lactam antibiotic use are indicated in Table 1. Some patients had more than one symptom. On chart review, patients with reactions to beta-lactam antibiotics other than penicillin were also identified. Of the 240 patients, there were 17 patients who described reactions to amoxicillin/ampicillin, 10 to cephalosporins, 4 to Cloxacillin, 3 to Piperacillin/tazobactam. All other patients reported “penicillin allergy”, whether this meant penicillin was the specific antibiotic causing reaction or a penicillin derivative is unknown.

**Table 1:** Reactions to beta-lactams

	Percentage of patients
Dermatologic	
Rash	100
Pruritus	28
Hives	32
Pustular rash	1
Angioedema	
Oropharyngeal/facial	20
Other	16
Anaphylaxis	2
Gastrointestinal	10
Respiratory	11
Cardiovascular	
Lightheaded	3
Tachycardia	1
Peripheral paraesthesias	2
Change in mental status	3
Pallor	1
Change in temperature sensation	2
Other	15
Delayed	21
Not known by patient	39
Not mentioned in chart	17

There was 17/240 patients with positive skin tests and their characteristics and results can be found in Table 2. Sixteen of these patients had ImmunoCap^®^ performed and one was positive. Three of the 17 patients underwent challenge, because of borderline positive skin tests and one had a reaction during challenge. Overall, four patients were skin test positive to MDM and five patients had positive skin tests to Penicillin G. During the entire period of testing, eight patients were skin test positive to PPL, one of these was also positive to MDM and one reacted to Penicillin G. Two patients were skin test-positive to ampicillin.

**Table 2:** Patients diagnosed with penicillin allergy pre-challenge

A. Positive skin tests										
Patient	Age	Sex	Reaction	Skin prick test		Intradermal		Immunocap		Challenge
				+	–	+	–	PenG	PenV	
1	17	F	Previous positive skin test, reaction unknown		PPL, MDM	PPL	MDM	ND	ND	ND
2	44	F	Rash		PPL, MDM	MDM	PPL	–	–	ND
3	49	M	Delayed hives		PPL, MDM	PPL	MDM	–	–	ND
4	29	M	Facial swelling		PPL, MDM, AMP	MDM, AMP	PPL	–	–	+
5	25	F	Unknown		PPL, MDM	PPL	MDM	–	–	ND
6	46	M	Unknown	n/d	n/d	PPL	MDM	–	–	ND
7	78	F	Delayed prolonged rash	MDM	PPL, CLX		MDM, PPL, CLX	–	–	ND
8	53	F	Facial swelling			PPL, MDM, AMP		–	–	ND
9	71	F	Oral tingling		PPL, PG, AMP	PPL, AMP	PG	–	–	–
10	41	F	Anaphylaxis		PPL	PG	CLX, ANF	–	–	ND
11	72	F	Delayed rash			PPL (delayed)	PG	–	+	ND
12	29	M	Not known	PG				–	–	ND
13	56	F	Rash			AMP (delayed)	PPL, PG	–	–	–
14	60	M	Rash			PPL, PG	CFZ	–	–	ND
15	19	F	Delayed rash	PG	PPL, AMP			–	–	ND
16	46	F	Rash	AMP	PPL, PG			–	–	ND
17	40	F	Hives, throat tightness			PG, AMP	PPL	–	–	ND

B. Positive specific IgE to Penicillin G or V by ImmunCap^**®**^						
Patient	Age	Sex	Reaction	Skin testing negative to	Immunocap	
					PenG	PenV
1	43	F	Erythema, pruritus	PPL, PG	–	+
2	25	F	Rash	PPL, PG	–	+
3	77	F	Rash, face/lip swelling	PPL, PG	+	–

A Positive skin test, B positive specific IgE (if negative skin testing)

*PPL* benzyl penicilloyl-polylysine, *MDM* minor determinant mixture, *PG* Penicillin G, *ND* not done, *AMP* ampicillin, *CLX* Cloxacillin, *CFZ* ceftazidime, *ANF* cefazolin

Twelve of 222 skin test-negative patients who were challenged had positive reactions to challenge. Information on these patients and their challenge reaction is included in Table 3, excluding the patient who had a positive challenge after a mildly positive skin test (Table 2A patient 4). Three of 12 patients who were positive to challenge were tested with Pen G and not MDM. Nine of 12 reacting to challenge had skin testing with MDM. It important to note that the challenge of three of the patients was inconclusive, because the physician supervising was unsure if the reaction that the patients complained of was due to the oral antibiotic. All three patients were to return for a blinded challenge but they did not attend the appointment. If these three patients are excluded, six of nine patients reacting to challenge had skin testing with MDM and three had testing with Penicillin G. Therefore, the false negative rate for testing with PPL and Penicillin G was 3/133 (2.3 %), NPV 97.74 %; CI 93.55–99.53 % and for testing with PPL and MDM was 6/86 (6.97 %), NPV 93.02 %; CI 85.43–97.4 % Chi square 2.954 p = 0.0856. The false negative rate for testing with all reagents (PPL, MDM and Penicillin G) was 5.4 %, NPV 94.6 %; CI 90.8–96.9 %.

**Table 3:** Patients with positive challenge

Patient	Age	Sex	Reaction	SPT/ID done	Challenge to	Challenge reaction
1	31	F	Pruritus and facial rash, hand swelling, dyspnea	SPT/ID-PPL, MDM, AMP	PenV	Pruritus, heavy chest, arm rash
2	42	F	Pruritic rash, throat itch	SPT/ID-PPL, MDM	PenV	Generalized pruritus
3	38	F	Rash	SPT/ID-PPL, MDM	Pen V	Next morning facial swelling—inconclusive
4	19	F	Urticaria	SPT/ID-PPL, MDM	PenV	Maculopapular rash forearm and hand, nasal and throat pruritus. Delayed -> non IgE mediated penicillin allergy
5	40	F	Angioedema tongue	SPT/ID-PPL, MDM	PenV	Pruritus, eye swelling
6	50	F	Urticaria	SPT-PPL, MDM	PenV	n/a
7	57	F	Shortness of breath	SPT-PPL, MDM	PenV	Pruritus, nausea—inconclusive
8	30	F	Rash	SPT/ID-PPL, MDM	PenV	Lip tingling, pruritic macular rash on chest, facial and back pruritus
9	47	F	Unknown, previous +	ID-PPL, PG	PenV	Throat sensation—inconclusive
10	50	F	Hives	ID-PPL, PG, AMP	PenV	Erythematous pruritus, palmar erythema
11	72	M	Rash	ID-PPL, PG, AMP	Amox	Delayed pruritic rash
12	32	F	Pruritus, hives, throat closing, lip swelling, paraesthesia	ID-PPL, PG, AMP	Amox	Pruritus to back and arms

## Discussion

The most common drug “allergy” reported in Europe and North America is to penicillin or its derivatives, with amoxicillin being the most common antibiotic prescribed in this class [1, 2]. However, the ability of patients to identify themselves as “penicillin allergic” does not correlate clinically with clinical IgE mediated immediate reactions [3, 4].

The lack of availability of commercial reagents required to properly test for an IgE mediated reaction, according to current guidelines, has theoretically made diagnosing penicillin allergy a challenge in current practice [6, 9].

Important symptoms to note that suggest immediate reactions are urticaria, angioedema, gastrointestinal symptoms, respiratory symptoms, hypotension and anaphylaxis [5, 6, 8]. However, for many patients, their reaction was a number of years ago and the clinical history is vague or unknown. Even if the patient’s history is not consistent with immediate hypersensitivity, skin testing may still be used to confirm absence of a life threatening reaction prior to challenge and is reassuring to the patient. However, it is important to note that negative skin testing results with a complete battery of penicillin skin test reagents does not always rule out an immediate allergy to penicillin [4, 21–23]. Patients should have a full workup including skin testing and challenge in order to rule out immediate hypersensitivity to penicillin as completely as possible, including, when possible, testing with the beta-lactam that precipitated the reaction.

The standard of practice for skin testing includes the use of PPL and MDM. It was previously thought that testing without a complete panel of minor antigenic determinants could fail to identify 10 % or more of patients with penicillin allergy [24–27]. When MDM are not available, Penicillin G has been used as an alternative, with PPL. Evidence for this approach comes from a study by Macy and Ngor [28] where they showed that 1125 patients would have to be skin tested with minor determinants to prevent one patient having a positive oral challenge with penicillin. This data resulted in these investigators discontinuing the use of penicilloate and penilloate (as components of MDM) skin testing for diagnosis of penicillin allergy. They concluded that it is possible to diagnose IgE mediated penicillin allergy using skin testing to penicilloyl-polylysine and penicillin and if negative, subsequent oral challenge with amoxicillin. Macy reports the result of skin testing with PPL (Prepen, ALK-Abello, USA) Penicillin G and amoxicillin [18, 28]. Using this method there were no anaphylactic reactions for skin test negative patients undergoing challenge and only four patients had minor reactions (hives within 1 h) with challenge.

Another study by del Real et al. [29] also used a skin testing protocol with PPL and Pen G in both the outpatient and inpatient (including ICU) setting. About half of those patients, who were negative on testing, were treated with beta-lactam antibiotics and less than 2 % had adverse reactions. These reactions were usually rash, with most of them being delayed reactions. The studies by Macy and by Del Real show the negative predictive value of using only PPL and Pen G is similar to that of a full panel with PPL and MDM [28–30].

Even more striking were the results of Picard et al. [31], who tested 563 children with a history of penicillin allergy only with Pen G, at a time when the PPL determinant was not available commercially in North America. They found that 32 % were positive to skin testing with the Pen G preparation, and challenged the skin test negative group with the incriminated beta-lactam or amoxicillin. Only 4.8 % reacted to challenge, with a NPV of 95.2 % (95 % CI 92.5–97.1 %).

These studies give results similar to our results, where we found only three positive challenges out of 133 patients challenged who were skin test negative to Penicillin G and PPL. Of 86 patients who were skin test negative to PPL and MDM, there were six positive challenges. None of these patients had severe reactions, as seen in Table 3. The false negative rate for testing with PPL and Penicillin G was 3/130 (2.3 %), NPV 97.74 %; CI 93.55–99.53 % and for testing with PPL and MDM was 6/86 (6.97 %), NPV 93.02 %; CI 85.43–97.4 % Chi square 2.954 p = 0.0856.

In contrast to Macy’s findings, a previous retrospective review done by Lin et al. [27] shows that by eliminating penicilloate and penilloate as components of the MDM one would fail to detect 16 % of patients with penicillin allergy. A minor determinant consisting of penilloate, penicilloate and Penicillin G will find 22.6 % of penicillin allergic patients vs. 6.6 % with Penicillin G alone. In this study, patients with negative skin tests were not challenged. Therefore, it would have been expected that a larger number of patients would be challenge positive after our practice protocol shifted from using MDM to Penicillin G. However, it is possible that the results of Lin et al. reflect a difference in the time since reaction between their patient population and our population. The longer the time since the reaction, the less likely it is that positive skin tests will be found [32, 33].

Overall we diagnosed 30 patients (12.55 %) with penicillin allergy, either by skin tests, ImmunoCap^®^ or challenge, which is in line with previous recent studies of patients with a history of penicillin allergy [17, 34]. Of the 521 patients whose charts were reviewed, 205 patients were skin test and ImmunoCap^®^ negative but were not challenged because they failed to return to the clinic for challenge.

Shortfalls of our study include the large number of patients who did not return for challenge. We do not know what the challenge results would be for the 205 patients who were skin test and ImmunoCap^®^ negative. We do not know if those patients who were challenge positive without skin testing to MDM would have been MDM skin test positive.

In future, if patents were challenged immediately after skin testing as in the protocol outlined by Macy, patients’ compliance would not be a factor. The delay in challenging was in part due to time constraints and in part due to the time it took for the ImmunoCap^®^ results to become available.

In conclusion, we do not appear to have failed to diagnose penicillin allergy in patients who presented with suspected reactions to these antibiotics by not testing with the minor determinant mixture. Although we did have positive challenges in patients who were tested only with Penicillin G, this number was less than the number of positive challenges when testing with the complete MDM mixture was done, although the difference was not statistically significant. In addition, no challenge produced a clinically serious reaction.
